# Total Alkaloids from Bamboo Shoots and Bamboo Shoot Shells of *Pleioblastus amarus* (Keng) Keng f. and Their Anti-Inflammatory Activities

**DOI:** 10.3390/molecules24152699

**Published:** 2019-07-24

**Authors:** Yan Ren, Yisha Ma, Zhidan Zhang, Liying Qiu, Huanhuan Zhai, Ruimeng Gu, Yaping Xie

**Affiliations:** 1 College of Pharmacy, Southwest Minzu University, Chengdu 610041, China; 2 Tianjin Institute of Industrial Biotechnology, Chinese Academy of Sciences, Tianjin 300308, China; 3 SCIEX China, Beijing 100017, China

**Keywords:** *Pleioblastus amarus* (Keng) Keng f., total alkaloids, LC-MS/MS, anti-inflammatory activity, RAW264.7 cells, ERK signaling

## Abstract

The bamboo shoot of *Pleioblastus amarus* (Keng) Keng f. is a medicinal and edible plant product in China. In this study, the chemical composition of the total alkaloids from bamboo shoots and bamboo shoot shells of *P. amarus* (Keng) Keng f. (ABSP and ABSSP, respectively) were separated and investigated by UHPLC/QTOF-MS/MS. The results showed that a total of 32 alkaloids were extracted, with 15 common to both ABSP and ABSSP and 10 and 7 alkaloids distinct to ABSP and ABSSP, respectively. ABSP and ABSSP both decreased the lipopolysaccharide (LPS, 0.5 μg/mL)-induced nitric oxide (NO) production in RAW264.7 murine macrophages with half maximal inhibitory concentration (IC_50_) values of 78 and 55 μg/mL, respectively. We also found that ABSP and ABSSP (100 μg/mL) could decrease the expression of inducible nitric oxide synthase (iNOS) and cyclooxygenase-2 (COX-2) at both mRNA and protein levels in LPS-exposed RAW264.7 cells. Moreover, 100 μg/mL of ABSP and ABSSP also significantly inhibited LPS-induced mRNA expression of interleukin 1β (IL-1β) and tumor necrosis factor α (TNF-α). Additionally, ABSP and ABSSP (100 μg/mL) decreased the phosphorylation of extracellular regulated protein kinase (ERK) in LPS-stimulated RAW264.7 cells. Collectively, the total alkaloids from the bamboo shoots and shells of *P. amarus* exhibit anti-inflammatory effects in LPS-activated RAW264.7 cells through the inhibition of ERK signaling. This result can provide support for the medicinal use and further study of *P. amarus*.

## 1. Introduction

Inflammation is not only the most primitive protective response to various stimuli, but also a common pathophysiological occurrence in a variety of diseases, such as cancer, diabetes, and rheumatoid arthritis [1]. It always starts with signal recognition and involves a series of reactions, including the transcription and translation of proinflammatory cytokines and inducible enzymes as well as the production of nitric oxide (NO) and prostaglandin E2 (PGE2) [2]. Several signaling cascades, such as the mitogen-activated protein kinases (MAPKs) and the nuclear factor-kappa B (NF-κB) pathways, play critical roles in the initiation and progression of inflammation [3,4,5,6,7,8]. Therefore, targeting these inflammatory mediators and signaling pathways may be a feasible strategy to prevent inflammatory diseases.

Recently, there has been growing interest in functional foods because of their potential positive effects on health beyond the original nutritional value [9,10]. The beneficial effects of these foods can be attributed to their bioactive compounds, such as flavonoids, polysaccharides, and alkaloids. Alkaloids are one of the largest groups of bioactive compounds, with great chemical diversity and various pharmacological properties, including anti-inflammatory [11], antiproliferative [12], antiangiogenic [13], antioxidant [14], and several other activities [15,16]. There has been great progress recently in the discovery of alkaloids with anti-inflammatory properties. For example, the alkaloid solanine A, isolated from the medicinal and edible plant *Solanum nigrum* Linn., exhibits potent anti-inflammatory effects in vitro and in vivo [17,18]. Thus, alkaloids from plants or functional foods may be considered as potential drugs for treating inflammatory diseases. The bamboo shoot is not only a traditional food ingredient, but has also been used in folk medicine for the treatment of dysentery, diarrhea, diabetes, influenza, and infection throughout history in China, such as ‘diet therapy herbs’ in the Tang dynasty [19]. Previous phytochemical studies revealed the presence of flavonoids, proteins, dietary fiber, alkaloids, and lignin in both bamboo shoots and bamboo shoot shells [20,21,22]. Recently, the bamboo shoot has attracted tremendous attention because of its high medicinal value and prospects for its development and utilization as an agent with antioxidative, anti-inflammatory, antibacterial, hepatoprotective, and hypolipidemic properties [23,24,25,26]. Although several bioactive components and pharmacological properties of bamboo shoots and shells have been reported previously, the bioactivities and the underlying mechanisms of its alkaloids have not yet been clearly elucidated. To further explore the medicinal value of alkaloids in bamboo shoots and bamboo shoot shells, we investigated the total alkaloid content of the bamboo shoots (abbreviation: ABSP) and bamboo shoot shells (abbreviation: ABSSP) of *Pleioblastus amarus* (Keng) Keng f. and identified their structures. Furthermore, the anti-inflammatory properties of the total alkaloids from bamboo shoots and shells were assessed in lipopolysaccharide (LPS)-activated RAW264.7 cells, and the underlying mechanisms were explored.

## 2. Results and Discussion

### 2.1. Identification of Alkaloids

**Figure 1:**
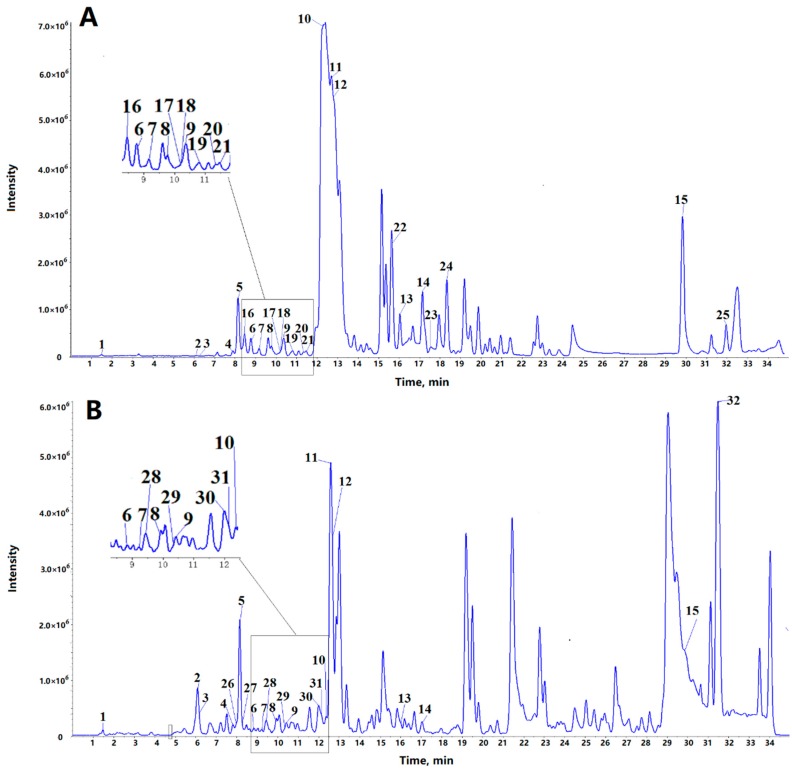
Total ion chromatogram of alkaloids from ABSP (**A**) and ABSSP (**B**) by LC-MS/MS. A total of 32 alkaloids were identified and the corresponding numbers listed in Table 1. ABSP: bamboo shoots of *Pleioblastus amarus* (Keng) Keng f.; ABSSP: bamboo shoot shells of *P. amarus* (Keng) Keng f.

**Figure 2:**
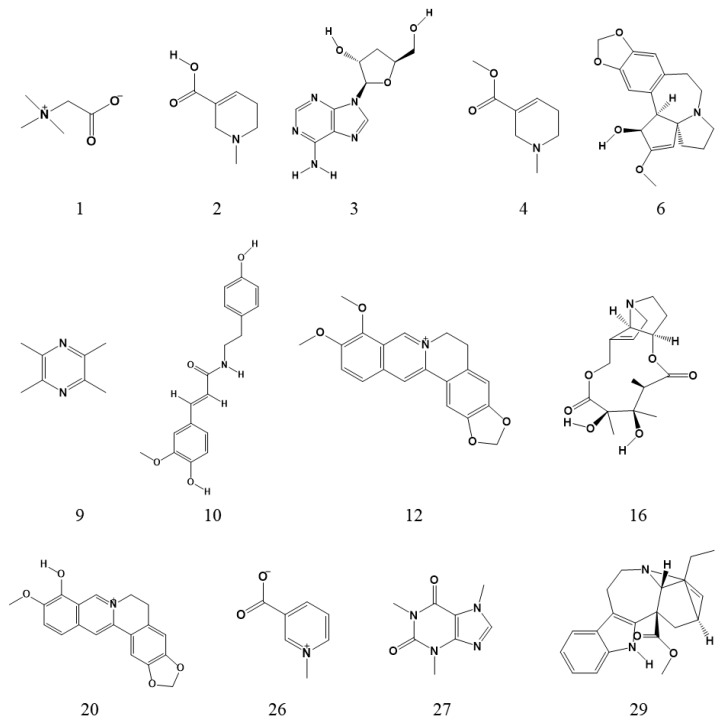
The chemical structures of the main alkaloids identified in ABSP and ABSSP. The numbers of the chemical structures correspond with those listed in Table 1. (1) Betaine; (2) arecaidine; (3) cordycepin; (4) arecoline; (6) cephalotaxine; (9) ligustrazine; (10) N-feruloyltyramine; (12) berberine; (16) monocrotaline; (20) berberrubine; (26) trigonelline; (27) caffeine; (29) catharanthine.

Alkaloids were analyzed, and their chemical compositions were determined by ultrahigh-performance liquid chromatography–quadrupole time-of-flight tandem mass spectrometry (UHPLC/QTOF-MS/MS). The total ion chromatograms are shown in Figure 1. In this study, most of the compounds showed a higher response in the positive ion mode than in the negative ion mode. Therefore, the positive mode was employed to identify the corresponding signals. The identification of 13 alkaloids was carried out by comparing their retention times, accurate mass, and MS/MS spectra with authentic standards. Their chemical structures are shown in Figure 2. One compound (No. 14 in Table 1) was characterized by comparing accurate mass and MS/MS spectra with data from a previous report [27]. The remaining compounds, for which no commercial standard was available or reported in literature, were tentatively characterized based on accurate mass and MS/MS fragments, especially using characteristic fragment ions that include nitrogen.

A total of 32 alkaloids in the bamboo shoots and shells were identified according to accurate mass and the characteristic fragments at low and high collision energy. Table 1 shows the retention time, name, and characteristic fragment ions of the identified compounds. The bamboo shoots contained 25 alkaloids, and the bamboo shoot shells contained 22 alkaloids. The bamboo shoots and bamboo shoot shells shared 15 alkaloids, while the bamboo shoots contained 10 distinct alkaloids and the bamboo shoot shells contained 7 distinct alkaloids.

### 2.2. ABSP and ABSSP Inhibit LPS-Induced Nitric Oxide Production in RAW264.7 Cells

**Figure 3:**
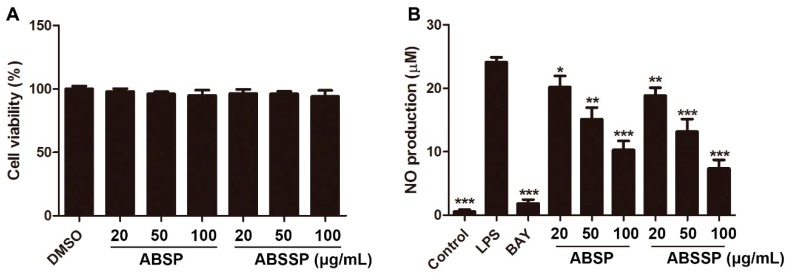
The effects of ABSP and ABSSP on cell viability and nitric oxide (NO) production in lipopolysaccharide (LPS)-stimulated RAW264.7 cells. (**A**) RAW264.7 cells were treated with different concentrations of ABSP or ABSSP for 24 h in the presence of LPS (0.5 μg/mL). Cell viability was assessed using the Cell-Counting Kit-8 (CCK8) method and expressed relative to the vehicle control. (**B**) RAW264.7 cells were pretreated with the indicated concentrations of ABSP or ABSSP and BAY11-7082 (BAY, 5 μM) for 2 h, and then exposed to LPS for an additional 24 h. Levels of NO in the culture medium were detected using Griess reagent. Data are expressed as mean ± SD, *n* = 3. * *p* < 0.05, ** *p* < 0.01, *** *p* < 0.001, compared with LPS.

To test the anti-inflammatory effects of total alkaloids from bamboo shoots and bamboo shoot shells, we used RAW264.7 macrophages as models. To eliminate the interference of cytotoxic effects of ABSP and ABSSP on the detection of anti-inflammatory activity, we measured the effects of different concentrations of ABSP and ABSSP on the cell viability of RAW264.7 macrophages in the presence of LPS. Results show that treatment with ABSP or ABSSP (0–100 μg/mL) did not affect the viability of RAW264.7 cells (Figure 3A). Thus, nontoxic concentrations from 20 to 100 μg/mL were used in the next studies.

To detect whether ABSP and ABSSP exhibited anti-inflammatory activity, we measured the effects of ABSP and ABSSP on LPS-induced release of nitric oxide (NO), a crucial inflammatory biomarker. Exposure to immunologic and inflammatory stimuli results in an overproduction of NO, which can trigger tissue injury via lipid peroxidation and DNA damage and cause chronic inflammation by upregulating the release of proinflammatory cytokines [28]. Thus, NO has been implicated as a pathogenic mediator in a variety of inflammatory diseases, such as rheumatoid arthritis (RA), Alzheimer’s disease, and inflammatory bowel diseases (IBD) [29,30]. As shown in Figure 3B, both ABSP and ABSSP (20–100 μg/mL) dose-dependently suppressed LPS-induced NO production in RAW264.7 cells (*p* < 0.05–0.001); the inhibition rates of ABSP (100 μg/mL) and ABSSP (100 μg/mL) on NO production were 57% and 69%, respectively. The positive control BAY11-7082 also showed a potent inhibition of LPS-induced NO release in RAW264.7 cells, and the inhibition rate of BAY11-7082 at 5 μM was 92%. Therefore, both ABSP and ABSSP exhibited potent anti-inflammatory activities in vitro.

### 2.3. ABSP and ABSSP Suppress the mRNA Expression of Proinflammatory Cytokines in LPS-Exposed RAW264.7 Cells

**Figure 4:**
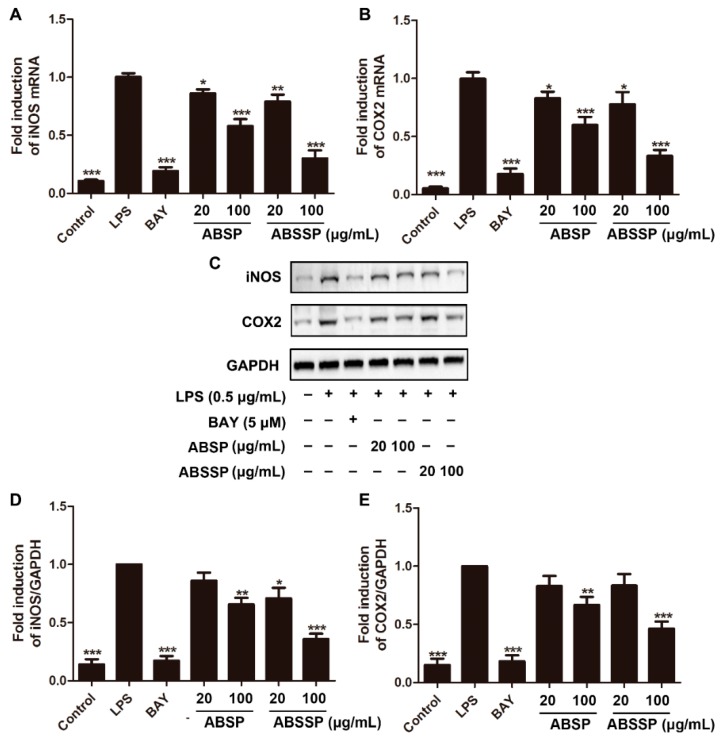
Effects of ABSP and ABSSP on mRNA and protein expression of inducible nitric oxide synthase (iNOS) and cyclooxygenase 2 (COX2) in LPS-stimulated RAW264.7 macrophage cells. RAW264.7 cells were pretreated with ABSP, ABSSP (20 and 100 μg/mL), and BAY11-7082 (BAY, 5 μM) for 2 h, and then stimulated with LPS (0.5 μg/mL) for 24 h. The mRNA levels of iNOS (**A**) and COX2 (**B**) were measured by qRT-PCR; Glyceraldehyde-3-phosphate dehydrogenase (GAPDH) was used as an internal control. Cell lysates were immunoblotted with anti-iNOS and anti-COX2 antibodies. GAPDH was used as a loading control. Results from representative experiments are shown (**C**), and the quantitative results of iNOS (**D**) and COX2 (**E**) are depicted. Data are expressed as mean ± SD, *n* = 3. * *p* < 0.05, ** *p* < 0.01, *** *p* < 0.001, compared with LPS.2.4. ABSP and ABSSP decreased the mRNA expression of interleukin 1β (IL-1β) and tumor necrosis factor α (TNF-α) in LPS-activated RAW264.7 cells.

The inducible nitric oxide synthase (iNOS) enzyme is responsible for the overproduction of NO under immunogenic and inflammatory stimuli [31]. We thus investigated whether the inhibitory effects of ABSP and ABSSP on NO production were due to their ability to inhibit iNOS expression. Figure 4A shows that 20 and 100 μg/mL ABSP decreased the mRNA levels of iNOS in LPS-stimulated RAW264.7 cells by 14% and 42%, respectively, while treatment with 20 and 100 μg/mL ABSSP resulted in 21% and 70% reduction in iNOS mRNA levels (*p* < 0.05–0.001), respectively. Consistent with their effects on iNOS transcription, repression of iNOS protein expression was observed in RAW264.7 cells treated with 100 μg/mL of ABSP and ABSSP by 34% and 64% (*p* < 0.01–0.001; Figure 4C,D), respectively. Cyclooxygenase 2 (COX2) is another key inducible enzyme that catalyzes the first and rate-limiting step in the formation of prostaglandin E2 (PGE2), which is an important proinflammatory biomarker [32]. Hence, COX2 is an attractive target for the development of anti-inflammatory agents [33]. As shown in Figure 4B, 20 and 100 μg/mL of ABSP or ABSSP diminished the transcription of COX2 into mRNA in LPS-exposed RAW264.7 macrophages by 17% and 40% or 22% and 67% (*p* < 0.05–0.001), respectively. Furthermore, treatment with 100 μg/mL ABSP or ABSSP reduced the LPS-induced protein expression of COX2 in RAW264.7 cells by 33% and 54% (*p* < 0.01–0.001), respectively. The resulting efficacy of BAY11-7082 (5 μM) in reducing iNOS and COX2 expression was more potent than that of ABSP or ABSSP. Taken together, ABSP and ABSSP exhibited anti-inflammatory activities by suppressing the expression of iNOS and COX2 at both transcription and translation levels.

**Figure 5:**
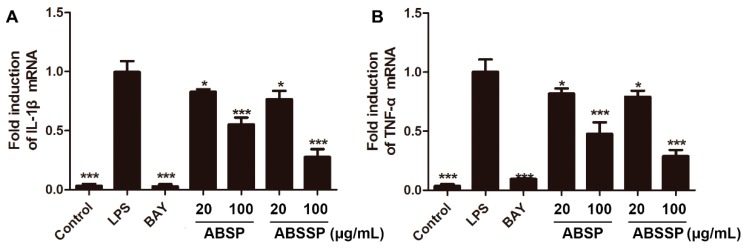
Effects of ABSP and ABSSP on the mRNA expression of IL-1β and TNF-α in LPS-exposed RAW264.7 cells. Cells were pretreated with different concentrations of ABSP, ABSSP (20 and 100 μg/mL), and BAY11-7082 (BAY, 5 μM) for 2 h, and then stimulated with LPS (0.5 μg/mL) for an additional 24 h. The mRNA levels of IL-1β (**A**) and TNF-α (**B**) were tested by a qRT-PCR method. GAPDH was used as an internal control. Data are expressed as mean ± SD, *n* = 3. * *p* < 0.05, ** *p* < 0.01, *** *p* < 0.001, compared with LPS.

Various inflammatory stimuli, including LPS, cause the transcription and translation of proinflammatory cytokines, leading to the initiation and amplification of the inflammatory response [34]. We therefore assessed whether ABSP and ABSSP can alter the mRNA levels of interleukin 1β (IL-1β) and tumor necrosis factor α (TNF-α) in LPS-exposed RAW264.7 cells. At doses of 20 and 100 μg/mL, both ABSP and ABSSP obviously downregulated the mRNA expressions of IL-1β and TNF-α, as did BAY11-7082 (Figure 5A,B). The inhibition rates of 20 and 100 μg/mL ABSP on IL-1β mRNA expression were 17% and 44% and were 18% and 52% on TNF-α mRNA levels (*p* < 0.05–0.001), respectively. Treatment with 20 and 100 μg/mL ABSP decreased the mRNA levels of IL-1β by 23% and 72%, respectively, and of TNF-α by 21% and 71%, respectively, compared with the LPS control (*p* < 0.05–0.001). IL-1β and TNF-α, two major proinflammatory cytokines, promote the inflammatory response both locally and systemically in many kinds of acute and chronic inflammatory diseases [35]. Therefore, the suppression of IL-1β and TNF-α is beneficial for the treatment of inflammation-related diseases [36]. The present data suggest that the anti-inflammatory effects of ABSP and ABSSP are due, at least in part, to their suppression of the expression of IL-1β and TNF-α at the transcription level.

### 2.4. ABSP and ABSSP Block LPS-Induced ERK Activation in RAW264.7 Cells

**Figure 6:**
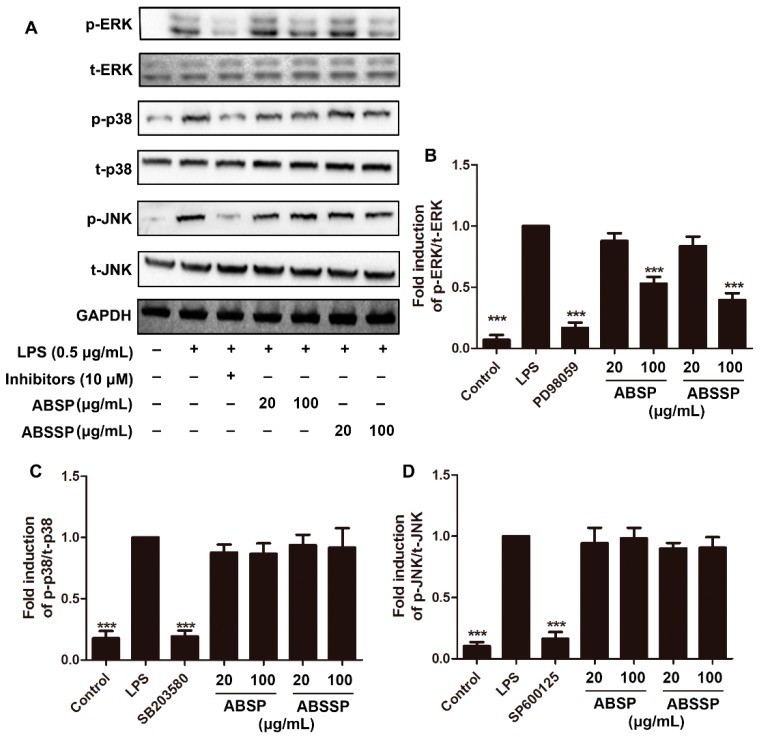
Effects of ABSP and ABSSP on LPS-induced activation of extracellular regulating kinase (ERK), p38 mitogen-activated protein kinase (MAPK), and c-Jun N-terminal kinase (JNK) in RAW264.7 cells. RAW264.7 cells were pretreated with different concentrations of ABSP and ABSSP or specific inhibitors (PD98059, SB203580, and SP600125; 10 μM) for 4 h, and then exposed to LPS for an additional 30 min. Next, cell lysates were used to detect the phosphorylation levels of ERK, JNK, and p38 MAPK using specific antibodies. The nonphosphorylated MAPK proteins were used as the loading control. Results from representative experiments are shown (**A**). Quantification of the ratio of phosphorylated ERK (**B**), p38 MAPK (**C**), and JNK (**D**) normalized to the loading control. Data are expressed as mean ± SD, *n* = 3. *** *p* < 0.001, compared with LPS.

MAPKs are evolutionarily conserved kinases that play pivotal roles in signal transduction mediated by cytokines, growth factors, and various environmental stresses [37]. In mammals, the MAPK family include three distinct members: the extracellular regulating kinase (ERK), p38 MAPK, and the c-Jun N-terminal kinase (JNK) [37]. A growing number of studies suggest that MAPKs play pivotal roles in the release of proinflammatory mediators and in downstream signaling events leading to acute or chronic inflammatory responses [38]. LPS can trigger the phosphorylation of MAPKs by binding and activating Toll-like receptor 4 (TLR4) in macrophages [39]. We therefore determined whether ABSP and ABSSP alters the LPS-induced activation of MAPK signaling, thereby inhibiting the inflammatory response. As shown in Figure 6, ERK, p38 MAPK, and JNK were phosphorylated in RAW264.7 cells after treatment with LPS. Inhibitors of ERK, p38 MAPK, and JNK, which were PD98059, SB203580, and SP600125 (10 μM each), respectively, attenuated the phosphorylation of the corresponding kinases by up to about 80% (*p* < 0.001). Pretreatment with 100 μg/mL ABSP or ABSSP significantly decreased the phosphorylation of ERK by 47% and 60% (*p* < 0.001), respectively. In contrast, LPS-induced activation of p38 MAPK and JNK1/2 in RAW264.7 cells were not affected by treatment with ABSP or ABSSP (*p* > 0.05). These results show that the inhibition by ABSP and ABSSP of LPS-induced activation of ERK may contribute to their anti-inflammatory potencies.

## 3. Materials and Methods

### 3.1. Plant Materials and Extract of Alkaloids

The bamboo shoots of *P. amarus* (Keng) Keng f. were collected from Leshan, Sichuan Province, China. The bamboo shoots were harvested in May. The plant sample was authenticated by Xianming Lu (College of Pharmacy, Chengdu University of Traditional Chinese Medicine, China).

The crude ethanol extracts from dried bamboo shoots and bamboo shoot shells of *P. amarus* (Keng) Keng f. were obtained by treatment with 95% ethanol at 25 °C. After the solvent was evaporated under reduced pressure, the crude ethanol extract was mixed with 1% HCl and then extracted with CHCl_3_. The total alkaloids from bamboo shoots (ABSP) and bamboo shoot shells (ABSSP) of *P. amarus* (Keng) Keng f. were obtained after solvent evaporation and drying.

### 3.2. Conditions of UHPLC/QTOF-MS/MS

Alkaloids were analyzed by a UHPLC LC-30A system (Shimadzu, Kyoto, Japan) equipped with an Xbridge BEH amide column (100 mm × 2.1 mm, 2.5 μm) (Waters, Milford, MA, USA) and coupled with a Triple TOF 5600 mass spectrometer (Sciex, Framingham, MA, USA). Mobile phase A was 10 mM ammonium acetate, and mobile phase B was 100% acetonitrile. The LC gradient was: 0–3 min, 95% B; 3–8 min, 95–90% B; 8–13 min, 80% B: 13–20 min, 65% B; 20–28 min, 50% B; 28–38 min, 50% B; and 38–40 min, 95% B. The flow rate was 0.2 mL/min. Ion voltage of electrospray ionization positive mode with a mass range of 50–1200 *m*/*z* was 5500 V. Gas 1 (nebulizer gas), gas 2 (heater gas), and curtain gas pressures were 55 psi, 55 psi, and 35 psi, respectively, and the source temperature was 600 °C. Acquisition of 15 MS/MS scans with a mass range of 30–1200 *m*/*z* was controlled by the information-dependent acquisition (IDA) function of the Analyst 1.6 software (Sciex, Framingham, MA, USA) with dynamic background subtraction on. The declustering potential was 80 V, and the collision energy was 35 ± 15 V. Calibration of mass accuracy was performed for every five samples. Data analysis for LC-MS/MS was performed with PeakView 2.0 software (Sciex, Framingham, MA, USA).

### 3.3. Drugs and Chemicals

LPS from *Escherichia coli* 055: B5 was obtained from Sigma (Shanghai, China). BAY 11-7082, PD98059, SB203580, SP600125, Cell-Counting Kit-8 (CCK-8), and Griess reagent were obtained from Beyotime (Haimen, China). Antibodies against glyceraldehyde 3-phosphate dehydrogenase (GAPDH), COX2, iNOS, total and phosphorylated ERK1/2, total and phosphorylated p-38 MAPK, and total and phosphorylated JNK were provided by SAB (Baltimore, MD, USA). 

### 3.4. Cell Culture

RAW264.7 cells were provided by the Cell Bank of the Chinese Academy of Sciences (Shanghai, China). Cells were cultured in Dulbecco’s Modified Eagle’s Medium (DMEM) supplemented with 10% fetal bovine serum (FBS) and 1% penicillin/streptomycin (Hyclone, Beijing, China) in atmosphere with 5% CO_2_ at 37 °C.

### 3.5. Determination of Cell Viability

Cell viability was measured by the CCK-8 reagent. Briefly, RAW264.7 cells (2.5 × 10^4^ cells/well) were seeded into a 96-well plate and incubated overnight at 37 °C. Next, cells were treated with different concentrations of ABSP or ABSSP for 24 h with LPS (0.5 μg/mL). Subsequently, 10 μL of CCK-8 were added into each well, and then incubated for 2 h at 37 °C. Next, absorbance at 450 nm was measured using a microplate reader (Thermo Fisher, Waltham, MA, USA). Relative cell viability was defined as the ratio of the absorbance in test wells compared to control wells.

### 3.6. Measurement of Nitric Oxide (NO)

Briefly, about 2.5 × 10^5^ of RAW264.7 cells were seeded into 24-well plates and incubated overnight. Cells were then treated with different concentrations of ABSP or ABSSP for 2 h, followed by stimulation of LPS (0.5 μg/mL) for an additional 24 h. Levels of NO in cell culture medium were evaluated using Griess reagent.

### 3.7. Quantitative Real-Time Polymerase Chain Reaction (qRT-PCR)

RAW264.7 cells (2 × 10^6^ cells/well) were seeded onto 6-well plates and incubated overnight at 37 °C. After treatment with different concentrations of ABSP or ABSSP and BAY 11-7082 for 2 h, cells were then exposed to LPS for an additional 24 h. Total RNA from each treatment was extracted using a UNLQ-10 Column total RNA Purification Kit (Sangon, Shanghai, China) according to the manufacturer’s instructions. Total RNA from the treatments were then reverse-transcribed to complementary DNA (cDNA) using the All-in-One cDNA Synthesis SuperMix Kit (Bimake, Shanghai, China). qRT-PCR was performed on a CFX96 Real-Time PCR System (Bio-Rad, Hercules, CA, USA) with SYBR Green (Bimake, Shanghai, China). Relative expression levels of the target genes were calculated based on 2^−ΔΔCt^ according to the manufacture’s specifications, using the GAPDH gene as a housekeeping gene. The primers used were as follows: TNF-α forward primer: 5′-CAC CAC GCT CTT CTG TCT-3′, TNF-α reverse primer: 5′-GGC TAC AGG CTT GTC ACT C-3′; IL-1β forward primer: 5′-CAA CCA ACA AGT GAT ATT CTC CAT G-3′, IL-1β reverse primer: 5′-GAT CCA CAC TCT CCA GCT GCA-3′; iNOS forward primer: 5′-CCT GTG AGA CCT TTG ATG-3, iNOS reverse primer: 5′-CCT ATA TTG CTG TGG CTC-3′; COX2 forward primer: 5′-CAA CAC CTG AGC GGT TAC-3′, COX2 reverse primer: 5′-GTT CCA GGA GGA TGG AGT-3′; GAPDH forward primer: 5′-TGC ACC ACC AAC TGC TTA GC-3′, GAPDH reverse primer: 5′-GGC ATG GAC TGT GGT CAT GAG-3′.

### 3.8. Western Blot Analysis

After specific treatments, RAW264.7 cells were lysed in radio-immunoprecipitation assay buffer containing 1% protease and phosphatase inhibitors (Bimake, Shanghai, China). The cell lysates were then separated using sodium dodecyl sulphate polyacrylamide gel electrophoresis (SDS-PAGE). Next, the proteins were blotted onto the polyvinylidene fluoride membrane (Millipore, Bedford, MA, USA). The membrane was blocked with 5% nonfat milk in Tris-buffered saline with Tween-20 (TBST) and incubated with the relevant primary antibodies at 4 °C overnight. After washing with TBST thrice, the membranes were incubated with horseradish peroxidase (HRP)-conjugated secondary antibody for 1 h at 25 °C, and then washed with TBST thrice. Signals were detected by chemiluminescence (Beyotime, Haimen, China), and quantified by using ImageJ software.

### 3.9. Statistical Analysis

All experiments were repeated at least 3 times, and representative results are presented. Data are expressed as mean values ± standard deviation (mean ± SD). Statistical analyses were performed with GraphPad Prism 5.0 software (GraphPad, La Jolla, CA, USA), and compared by analysis of variance (ANOVA) followed by a Tukey Test. The differences were considered statistically significant when *p* < 0.05.

## 4. Conclusions

In this work, we identified and characterized the alkaloids in bamboo shoots and bamboo shoot shells of *P. amarus* (Keng) Keng f. using UHPLC/QTOF-MS/MS. We found that the total alkaloids of bamboo shoots and bamboo shoot shells (ABSP and ABSSP, respectively) exhibited potent anti-inflammatory activity in LPS-exposed RAW264.7 macrophages by downregulating the production of proinflammatory mediators and cytokines. All these actions may be attributed to their ability to suppress the activation of the ERK signaling pathway. Furthermore, we observed that the anti-inflammatory activity of ABSSP was more efficient than that of ABSP in most of the tests. The differences in the intensity of anti-inflammatory effects of ABSP and ABSSP may be attributed to the different alkaloids derived from bamboo shoots and bamboo shoot shells. Additionally, these findings also suggest that bamboo shoots and shoot shells are valuable sources of alkaloids. Therefore, this study expands the applications of *P. amarus* (Keng) Keng f. as a functional substance.

## Figures and Tables

**Table 1:** Identification of alkaloids in the bamboo shoots and shells of *P. amarus* by UHPLC/QTOF-MS/MS in positive mode.

No.	Rt (min)	[M + H]^+^			Formula	Identification	MS/MS Fragments
		*m*/*z* theo	*m*/*z* exp	Error (ppm)			
Common alkaloids in the bamboo shoots and shells of *P. amarus*							
1	1.47	118.0863	118.0865	1.7	C_5_H_11_NO_2_	Betaine ^a^	100.076 (C_5_H_10_NO^+^), 82.065 (C_5_H_8_N^+^), 58.069 (C_3_H_8_N^+^)
2	6.15	142.0863	142.0863	0	C_7_H_11_NO_2_	Arecaidine ^a^	124.077 (C_7_H_10_NO^+^), 100.076 (C_5_H_10_NO^+^), 96.084, 69.035
3	6.23	252.1091	252.1089	−0.8	C_10_H_13_N_5_O_3_	Cordycepin ^a^	136.062 (C_5_H_6_N_5_^+^), 119.036 (C_5_H_3_N_4_^+^)
4	7.85	156.1019	156.1017	−1.3	C_8_H_13_NO_2_	Arecoline ^a^	138.093 (C_8_H_12_NO^+^), 110.103 (C_7_H_12_N^+^), 86.061, 69.037
5	8.07	238.1438	238.1439	0.4	C_13_H_19_NO_3_	- ^c^	164.107 (C_9_H_10_NO_2_^+^), 121.065, 103.055, 93.070, 91.055, 77.040
6	8.88	316.1543	316.1545	0.6	C_18_H_21_NO_4_	Cephalotaxine ^a^	283.041, 267.019, 169.098, 150.091, 133.064, 121.065, 103.054, 93.071, 91.054, 77.040
7	9.25	245.1648	245.1648	0	C_15_H_20_N_2_O	2-(4-Piperidinylcarbonyl)-1,2,3,4-tetrahydroisoquinoline ^b^	162.090 (C_10_H_12_NO^+^), 125.070 (C_6_H_9_N_2_O+), 70.067 (C_4_H_8_N^+^), 121.064, 103.054, 93.069, 91.055, 77.039
8	9.81	176.0706	176.0701	−2.8	C_10_H_9_NO_2_	- ^c^	158.059 (C_10_H_8_NO^+^), 130.065 (C_9_H_8_N^+^), 118.066 (C_8_H_8_N^+^), 128.049, 103.055, 91.055, 77.040
9	10.32	137.1073	137.1072	−0.7	C_8_H_12_N_2_	Ligustrazine ^a^	121.077 (C_7_H_9_N_2_^+^), 96.081 (C_6_H_10_N^+^), 70.065 (C_4_H_8_N^+^), 94.067, 80.051, 69.072, 55.057
10	12.35	314.1387	314.1393	1.9	C_18_H_19_NO_4_	N-feruloyltyramine ^a^	177.055, 149.060, 145.029, 121.066, 117.035, 103.055, 93.071, 91.055, 89.040, 77.041
11	12.72	227.0815	227.0816	0.4	C_13_H_10_N_2_O_2_	- ^c^	210.055 (C_13_H_8_NO_2_^+^), 199.050 (C_11_H_7_N_2_O_2_^+^), 198.055 (C_12_H_8_NO_2_^+^), 197.071 (C_12_H_9_N_2_O^+^), 181.076 (C_12_H_9_N_2_^+^), 169.076 (C_11_H_9_N_2_^+^), 154.065 (C_11_H_8_N^+^), 153.065 (C_7_H_9_N_2_O_2_^+^), 184.076, 144.080, 127.055, 115.055, 103.054, 77.039
12	12.75	336.1230	336.1232	0.6	C_20_H_17_NO_4_	Berberine ^a^	320.091 (C_19_H_14_NO_4_^+^), 306.076 (C_18_H_12_NO_4_^+^), 304.097 (C_19_H_14_NO_3_^+^), 292.097 (C_18_H_14_NO_3_^+^), 278.081 (C_17_H_12_NO_3_^+^), 262.086 (C_17_H_12_NO_2_^+^), 275.093
13	16.00	268.1332	268.1332	0	C_17_H_17_NO_2_	4-Acetyl-N-(2,6-dimethylphenyl)benzamide ^b^	174.094, 147.046, 131.049, 121.066, 119.051, 105.073, 103.055, 91.055, 79.057, 77.041, 65.041
14	17.03	386.1598	386.1598	0	C_21_H_23_NO_6_	Colchiceine ^b^ [27]	386.162 (C_21_H_24_NO_6_^+^), 368.149 (C_21_H_22_NO_5_^+^), 358.164 (C_20_H_24_NO_5_^+^), 342.134 (C_19_H_20_NO_5_^+^), 328.118 (C_18_H_18_NO_5_^+^), 326.139 (C_19_H_20_NO_4_^+^), 310.112 (C_18_H_16_NO_4_^+^), 162.089 (C_10_H_12_NO^+^), 308.131, 280.138, 179.071, 105.034
15	29.89	400.3210	400.3211	0.2	C_26_H_41_NO_2_	- ^c^	138.090 (C_8_H_12_NO^+^), 164.071, 121.064, 93.070
Alkaloids only in bamboo shoots of *P. amarus*							
16	8.24	326.1598	326.1601	0.9	C_16_H_23_NO_6_	Monocrotaline ^a^	150.091, 131.070, 121.065, 103.039, 85.029, 57.035
17	10.22	272.1281	272.1281	0	C_16_H_17_NO_3_	- ^c^	216.102 (C_13_H_14_NO_2_^+^), 194.081 (C_10_H_12_NO_3_^+^), 135.043, 121.067, 91.054
18	10.23	335.1750	335.1757	2.1	C_21_H_22_N_2_O_2_	- ^c^	188.107 (C_12_H_14_NO^+^), 215.112, 198.091, 121.065, 103.054, 95.061, 93.071, 91.055, 77.039
19	10.7	342.1340	342.134	0	C_19_H_19_NO_5_	Taspine’s derivate ^b^	310.108 (C_18_H_16_NO_4_^+^), 162.090 (C_10_H_12_NO^+^), 327.111, 292.097, 278.081, 266.081, 248.071, 220.0753, 189.078
20	11.42	322.1074	322.1074	0	C_19_H_15_NO_4_	Berberrubine ^a^	278.079 (C_17_H_12_NO_3_^+^), 307.085, 279.088, 264.065, 250.083
21	11.6	395.19653	395.1964	−0.3	C_23_H_26_N_2_O_4_	- ^c^	377.203 (C_23_H_25_N_2_O_3_^+^), 367.165 (C_21_H_23_N_2_O_4_^+^), 311.175 (C_19_H_23_N_2_O_2_^+^), 275.139 (C_15_H_19_N_2_O_3_^+^), 247.103 (C_13_H_15_N_2_O_3_^+^), 162.091 (C_10_H_12_NO^+^), 136.076 (C_8_H_10_NO^+^), 229.098, 155.080, 121.065
22	15.68	306.1700	306.1697	−1.0	C_17_H_23_NO_4_	- ^c^	260.131 (C_15_H_18_NO_3_^+^), 216.101 (C_13_H_14_NO_2_^+^), 181.112 (C_9_H_16_NO_3_^+^), 140.0706 (C_7_H_10_NO_2_^+^), 121.066, 113.026, 91.054
23	17.55	325.191	325.1911	0.3	C_20_H_24_N_2_O_2_	- ^c^	162.090 (C_10_H_12_NO^+^), 204.138, 203.154, 118.065, 105.033, 100.112, 77.039
24	18.5	370.129	370.1285	−1.4	C_20_H_19_NO_6_	Taspine ^b^	352.119 (C_20_H_18_NO_5_^+^), 342.135 (C_19_H_20_NO_5_^+^), 310.109 (C_18_H_16_NO_4_^+^), 162.090 (C_10_H_12_NO^+^), 327.111, 292.097, 278.082, 266.081, 248.069, 220.075, 189.078, 130.065, 105.033
25	31.81	430.3316	430.3322	1.4	C_27_H_43_NO_3_	- ^c^	166.087 (C_9_H_12_NO_2_^+^), 120.082 (C_8_H_10_N^+^)
Alkaloids only in bamboo shoot shells of *P. amarus*							
26	7.95	138.0550	138.0549	−0.7	C_7_H_7_NO_2_	Trigonelline ^a^	120.048 (C_7_H_6_NO^+^), 94.032 (C_6_H_8_N^+^), 82.068 (C_5_H_8_N^+^), 66.037 (C_4_H_4_N^+^), 65.042
27	8.27	195.0877	195.0875	−1	C_8_H_10_N_4_O_2_	Caffeine ^a^	138.067 (C_6_H_8_N_3_O^+^), 136.051 (C_6_H_6_N_3_O^+^), 110.072 (C_5_H_8_N_3_^+^), 123.042, 83.062, 69.047
28	9.46	328.1543	328.1544	0.3	C_19_H_21_NO_4_	- ^c^	208.098 (C_11_H_14_NO_3_^+^), 176.076 (C_10_H_10_NO_2_^+^), 273.112, 191.070, 121.065, 107.049, 103.055, 93.071
29	10.28	337.1911	337.1913	0.6	C_21_H_24_N_2_O_2_	Catharanthine ^a^	217.134, 121.065, 103.055, 97.077, 93.070, 77.039
30	11.99	353.1496	353.1495	−0.3	C_20_H_20_N_2_O_4_	N-Feruloylserotonin ^b^	160.076 (C_10_H_10_NO^+^), 221.105, 177.055, 145.028, 117.033, 89.039
31	12.24	323.1390	323.1393	0.9	C_19_H_18_N_2_O_3_	N-p-Coumaroylserotonin ^b^	229.097 (C_13_H_13_N_2_O_2_^+^), 159.092 (C_10_H_11_N_2_^+^), 130.065 (C_9_H_8_N^+^), 295.143, 278.117, 245.093, 172.076, 158.059, 131.051, 121.065, 107.051, 103.054, 93.070, 91.0560, 77.040
32	31.5	404.3159	404.3162	0.7	C_25_H_41_NO_3_	N-Palmitoyl-L-phenylalanine ^b^	166.086 (C_9_H_12_NO_2_^+^), 120.081 (C_8_H_10_N^+^), 149.059, 131.049, 103.055

^a^: Confirmation in comparison with authentic standards. ^b^: Characterized tentatively by accurate mass and MS/MS fragments. ^c^: Characterized tentatively by accurate mass and characteristic fragment ions including nitrogen.

## References

[1] Shi C., Yuan S., Zhang H., Zhang T., Wang L., Xu Z. (2010). Cell-mediated immune responses and protective efficacy against infection with Mycobacterium tuberculosis induced by Hsp65 and hIL-2 fusion protein in mice. Scand. J. Immunol..

[2] Deng J.-S., Huang G.-J., Huang S.-S., Wang S.-Y., Chang Y.-S., Kuo Y.-H. (2013). Bioassay Guided Isolation and Identification of Anti-inflammatory Active Compounds from the Root of Ficus formosana. J. Agric. Food Chem..

[3] Véronique B., Michael K. (2009). Is NF-κB a good target for cancer therapy? Hopes and pitfalls. Nat. Rev. Drug Discov..

[4] Leen C., Geert L.V. (2017). Inflammation and the Metabolic Syndrome: The Tissue-Specific Functions of NF-κB. Trends Cell Biol..

[5] Hua Y., Drew P., Richard J. (2009). STATs in cancer inflammation and immunity: A leading role for STAT3. Nat. Rev. Cancer.

[6] Martínez-Soto D., Ruiz-Herrera J. (2017). Functional analysis of the MAPK pathways in fungi. Rev. Iberoam. Micol..

[7] Matthews C.P., Colburn N.H., Young M.R. (2007). AP-1 a target for cancer prevention. Curr. Cancer Drug Targets.

[8] Yao B., Wang S., Xiao P., Wang Q., Hea Y., Zhang Y., He Y. (2017). MAPK signaling pathways in eye wounds: Multifunction and cooperation. Exp. Cell Res..

[9] Reis F.S., Martins A., Vasconcelos M.H., Morales P., Ferreira I.C. (2017). Functional foods based on extracts or compounds derived from mushrooms. Trends Food Sci. Technol..

[10] Wani T.A., Shah A.G., Wani S.M., Wani I.A., Masoodi F.A., Nissar N., Shagoo M.A. (2016). Suitability of Different Food Grade Materials for the Encapsulation of Some Functional Foods Well Reported for Their Advantages and Susceptibility. Crit. Rev. Food Sci. Nutr..

[11] Guo Y.-G., Ding Y.-H., Wu G.-J., Zhu S.-L., Sun Y.-F., Yan S.-K., Qian F., Jin H.-Z., Zhang W.-D. (2018). Three new alkaloids from Xylopia vielana and their antiinflammatory activities. Fitoterapia.

[12] Ma Y.L., Liu Y.P., Zhang C., Zhao W.H., Shi S., He D.N., Zhang P., Liu X.H., Han T.T., Fu Y.H. (2017). Carbazole alkaloids from Clausena hainanensis with their potential antiproliferative activities. Bioorg. Chem..

[13] Wei R., Ma Q., Li T., Liu W., Sang Z., Li M., Liu S. (2018). Carbazole alkaloids with antiangiogenic activities from Clausena sanki. Bioorg. Chem..

[14] Jiang X.-L., Wang L., Wang E.-J., Zhang G.-L., Chen B., Wang M.-K., Li F. (2018). Flavonoid glycosides and alkaloids from the embryos of Nelumbo nucifera seeds and their antioxidant activity. Fitoterapia.

[15] Lukhoba C.W., Simmonds M.S., Paton A.J. (2006). Plectranthus: A review of ethnobotanical uses. J. Ethnopharmacol..

[16] Salminen K.A., Meyer A., Jeřábková L., Korhonen L.E., Rahnasto M., Juvonen R.O., Imming P., Raunio H. (2011). Inhibition of human drug metabolizing cytochrome P450 enzymes by plant isoquinoline alkaloids. Phytomedicine.

[17] Gu X.-Y., Shen X.-F., Wang L., Wu Z.-W., Li F., Chen B., Zhang G.-L., Wang M.-K. (2018). Bioactive steroidal alkaloids from the fruits of Solanum nigrum. Phytochemistry.

[18] Zhao L., Wang L., Di S.-N., Xu Q., Ren Q.-C., Chen S.-Z., Huang N., Jia D., Shen X.-F. (2018). Steroidal alkaloid solanine A from Solanum nigrum Linn. exhibits anti-inflammatory activity in lipopolysaccharide/interferon γ-activated murine macrophages and animal models of inflammation. Biomed. Pharmacother..

[19] Changkija S. (1999). Folk Medicinal Plants of the Nagas in India. Asian Folk. Stud..

[20] Jin Y., Yuan K. (2012). Studies on the Functional Components and Bioactivity and the Relativity of Bamboo Shoots and Shells. Appl. Mech. Mater..

[21] Gong W., Xiang Z., Ye F., Zhao G. (2016). Composition and structure of an antioxidant acetic acid lignin isolated from shoot shell of bamboo (Dendrocalamus Latiforus). Ind. Crop Prod..

[22] Yang Y.F. (2009). A study on the flavonoid compound in bamboo shoots of three pleioblastus species. J. Bamboo Res..

[23] Luo X., Wang Q., Zheng B., Lin L., Chen B., Zheng Y., Xiao J. (2017). Hydration properties and binding capacities of dietary fibers from bamboo shoot shell and its hypolipidemic effects in mice. Food Chem. Toxicol..

[24] Zheng Y., Zhang S., Wang Q., Lu X., Lin L., Tian Y., Xiao J., Zheng B. (2016). Characterization and hypoglycemic activity of a beta-pyran polysaccharides from bamboo shoot (Leleba oldhami Nakal) shells. Carbohydr. Polym..

[25] Thomas R., Jebin N., Saha R., Sarma D. (2016). Antioxidant and antimicrobial effects of kordoi (Averrhoa carambola) fruit juice and bamboo (Bambusa polymorpha) shoot extract in pork nuggets. Food Chem..

[26] Zhang Y., Xu P., Xie Y.H., Du W.P., Jiang P., Yao Z.Z., Li B. (2013). Phyllostachys chemical and its resistance to liver damage components research. J. Anhui Agric. Sci..

[27] Hamscher G., Priess B., Nau H., Panariti E. (2005). Determination of Colchicine Residues in Sheep Serum and Milk Using High-Performance Liquid Chromatography Combined with Electrospray Ionization Ion Trap Tandem Mass Spectrometry. Anal. Chem..

[28] Soufli I., Toumi R., Rafa H., Touil-Boukoffa C., Soufli R.T.I. (2016). Overview of cytokines and nitric oxide involvement in immuno-pathogenesis of inflammatory bowel diseases. World J. Gastrointest. Pharmacol. Ther..

[29] Ali A.M., Habeeb R.A., El-Azizi N.O., Khattab D.A., Abo-Shady R.A., Elkabarity R.H. (2014). Higher nitric oxide levels are associated with disease activity in Egyptian rheumatoid arthritis patients. Rev. Bras. Reum..

[30] Asiimwe N., Yeo S.G., Kim M.S., Jung J.Y., Jeong N.Y. (2016). Nitric oxide: Exploring the contextual link with alzheimer’s disease. Oxid. Med. Cell. Longev..

[31] Aktan F. (2004). iNOS-mediated nitric oxide production and its regulation. Life Sci..

[32] Garret A., Fitz G. (2003). COX-2 and beyond: Approaches to prostaglandin inhibition in human disease. Nat. Rev. Drug Discov..

[33] O’Callaghan G., Houston A. (2016). Prostaglandin E2 and the EP receptors in malignancy: Possible therapeutic targets?. Br. J. Pharmacol..

[34] Wojdasiewicz P., Poniatowski Ł.A., Szukiewicz D. (2014). The Role of Inflammatory and Anti-Inflammatory Cytokines in the Pathogenesis of Osteoarthritis. Mediat. Inflamm..

[35] Tracey K.J. (2010). The inflammatory reflex. Nature.

[36] Siebert S., Tsoukas A., Robertson J., McInnes I. (2015). Cytokines as Therapeutic Targets in Rheumatoid Arthritis and Other Inflammatory Diseases. Pharmacol. Rev..

[37] Wada T., Penninger J.M. (2004). Mitogen-activated protein kinases in apoptosis regulation. Oncogene.

[38] Broom O.J., Widjaya B., Troelsen J., Olsen J., Nielsen O.H. (2010). Mitogen activated protein kinases: A role in inflammatory bowel disease?. Clin. Exp. Immunol..

[39] Tai C.J., Chang S.J., Leung P.C., Tzeng C.R. (2013). ATP activates nuclear translocation of mitogen-activated protein kinases in human granulosa-luteal cells. J. Clin. Endocrinol. Metab..

